# Ratifying the efficacy and safety of intensive induction chemotherapy for acute myeloid leukaemia by the Australasian Leukaemia & Lymphoma Group consensus approach

**DOI:** 10.1111/imj.70010

**Published:** 2025-03-07

**Authors:** Aditya Tedjaseputra, Amanda Tey, Anastasios Nalpantidis, George Grigoriadis, Shaun Fleming, Shahla Vilcassim, Pasquale L. Fedele, Michael Sze Yuan Low, Paul Yeh, Michael Gilbertson, Ashwini Bennett, Gareth P. Gregory, Danielle Oh, Donna Gairns, Zane Kaplan, Sanjeev D. Chunilal, Susan Brown, Stephen Opat, Chong C. Chua, Jake Shortt

**Affiliations:** ^1^ Monash Haematology Monash Health Melbourne Victoria Australia; ^2^ Department of Haematology Guy's and St. Thomas’ NHS Foundation Trust London UK; ^3^ Cancer Genetics Laboratory, Department of Medical and Molecular Genetics King's College London London UK; ^4^ Pharmacy Department Monash Health Melbourne Victoria Australia; ^5^ Department of Medicine School of Clinical Sciences at Monash Health, Monash University Melbourne Victoria Australia; ^6^ Department of Haematology Alfred Health Melbourne Victoria Australia; ^7^ Department of Haematology Northern Hospital Melbourne Victoria Australia; ^8^ Division of Blood Cells and Blood Cancer Walter and Eliza Hall Institute of Medical Research Melbourne Victoria Australia

**Keywords:** acute myeloid leukaemia, *fms‐like tyrosine kinase 3*, midostaurin, idarubicin

## Abstract

**Background:**

After pharmaceutical benefits scheme approval of midostaurin for fms‐like tyrosine kinase 3 (*FLT3*)‐mutated acute myeloid leukaemia (AML) in 2018, the Australasian Leukaemia & Lymphoma Group (ALLG) proposed a consensus approach to AML induction with 7+3 chemotherapy (7 days of infusional cytarabine with three doses of anthracycline) to align with future clinical trial protocols.

**Aims:**

To determine the efficacy and safety of idarubicin‐based 7+3 induction ± midostaurin (per ALLG recommendations) in a real‐world, tertiary hospital setting.

**Methods:**

Data were prospectively collected for all patients assessed for front‐line AML treatment. Disease risk and response assessments were defined by European LeukaemiaNet 2017 guidelines. Efficacy and safety endpoints included complete remission (CR) rates, composite CR rates, event‐free survival (EFS), overall survival (OS), induction mortality, duration of cytopenias and intensive care unit (ICU) utilisation. Analysis was planned following completion of ≥50 inductions and 5‐year aggregated experience.

**Results:**

Between 2018 and 2023, 58 patients (median age 49 years) received 7+3 induction with CR and induction mortality rates of 88% (95% confidence interval (95% CI): 77–95%) and 1.7% (95% CI: 0–9%) respectively. At a median of 24.6 months of follow‐up, median OS was 17.6 months for adverse‐risk versus not reached for non‐adverse‐risk patients (*P* = 0.03). *FLT3*‐mutated patients demonstrated an 89% CR rate (95% CI: 67%–99%) with comparable 4‐year EFS (65%) and OS (68%) to *FLT3*‐wild‐type patients. Safety across 58 induction and 139 consolidation cycles was acceptable, with a single death and a 21% ICU admission rate (95% CI: 11%–33%) during induction.

**Conclusions:**

Idarubicin‐based 7+3 induction with contemporary supportive care yields good safety and CR rates, including in midostaurin‐treated *FLT3*‐mutated patients. Survival outcomes for adverse‐risk AML patients remain suboptimal.

## Introduction

Attainment of complete remission (CR) is a key treatment goal in newly diagnosed acute myeloid leukaemia (AML), allowing risk‐stratified consolidation with curative intent.^1^ Variations of the 7+3 regimen (7 days of infusional cytarabine with three doses of anthracycline), have been the mainstay of remission induction for >50 years.^2^ However, reported CR and induction mortality rates vary widely in the literature, with up to one in seven younger patients dying due to treatment failure or associated complications in ‘real‐world’ series (and worse outcomes in patients aged >60).^3^, ^4^ Recently, the safety of intensive therapy has improved with the provision of better supportive care, including anti‐fungal prophylaxis.^5^, ^6^ Meanwhile, attempts to improve CR rates by chemo‐intensification came at the expense of increased toxicity and did not improve outcomes for subgroups with adverse cytogenetics or mutational profiles.^7^, ^8^, ^9^, ^10^

*fms‐like tyrosine kinase 3* (*FLT3*) mutations are found in about 30% of newly diagnosed AML cases and convey poorer prognosis when treated with conventional chemotherapy.^11^ The Cancer and Leukemia Group B 10603 RATIFY trial established the addition of the FLT3 inhibitor midostaurin to daunorubicin‐based 7+3 as the new standard of care for *FLT3*‐mutated patients. This was based on an 8% improvement in 4‐year overall survival (OS) relative to placebo.^12^ Midostaurin received Australian pharmaceutical benefits scheme (PBS) recommendation for this indication in December 2018.

The Australasian Leukaemia & Lymphoma Group (ALLG) is a 50‐year‐old not‐for‐profit cooperative clinical trial research group that has conducted several studies attempting to optimise AML induction strategies.^6^, ^7^ Recognising that midostaurin approval would set a template for future AML trials utilising standard 7+3 induction,^12^ the ALLG AML scientific working party suggested adopting a RATIFY‐like approach for AML induction in 2018. This represented a pivot from previous ALLG protocols, which incorporated escalated cytarabine doses in induction and using anthracyclines in consolidation.^6^, ^7^ The recommendation also adopted a ‘double induction strategy’, where non‐adverse ELN risk patients not in CR after the first 7+3 would receive a second 7+3 cycle rather than immediate intensification. Today, with the approval of additional novel agents for front‐line AML management (e.g., venetoclax,^13^ gemtuzumab ozogamicin,^14^ CPX‐351^15^), the ALLG consensus approach has evolved into a ‘living treatment considerations document’ available to members online (www.allg.org.au).

Other features of the Australian treatment landscape distinct to the RATIFY protocol^12^ include: (i) substitution of daunorubicin with idarubicin as the anthracycline due to the higher local daunorubicin cost, (ii) routine use of mould‐spectrum azole anti‐fungal prophylaxis concurrent to midostaurin (where there is a major cytochrome P450 interaction) and (iii) extension of the upper age limit for intensive induction with midostaurin to patients >59 years old. In this context, we report the safety and efficacy of a RATIFY‐like approach in newly diagnosed AML patients, focussing on midostaurin‐treated patients.

## Methods

### Patients and treatment setting

Monash Health is a tertiary healthcare network comprising >2000 inpatient beds among five hospitals serving a population of approximately 1.3 million.^16^ Acute inpatient haematology services are centralised at a university‐affiliated academic centre. Prior to August 2018, intensive AML inductions had not been performed in the network since the year 2000. Concurrent to resumption of AML inductions, a database was prospectively curated to benchmark outcomes and assure quality of care (Monash Health human research ethics reference: RES‐21‐0000015Q‐72 713). Formal efficacy and safety analysis was planned after at least 50 inductions and 5 years of aggregated experience, thus defining the study period as between August 2018 and July 2023. Data were collected on all consecutive adult patients (age ≥ 18) presenting with AML.

### Diagnosis and risk stratification

Diagnoses were confirmed by bone marrow examination and classified by the 2017 World Health Organization classification of tumours of haemopoietic and lymphoid tissues (Revised 4th Edition).^17^ Cytogenetic and molecular testing were performed in all patients considered for intensive induction. *FLT3* internal tandem duplication (*FLT3*‐ITD) and tyrosine kinase domain (*FLT3*‐TKD) mutation detection was by fragment length analysis and capillary electrophoresis as described previously (typical sensitivity 10^−2^), with results generally available within 7 days of induction commencement. There was no minimum allelic ratio (AR) required for *FLT3* inhibitor access.^18^, ^19^ Risk stratification was by European LeukaemiaNet (ELN) 2017 criteria.^20^ Where cytogenetic and/or molecular data were incomplete (e.g., due to failed mitoses), patients were empirically classified as intermediate risk.

### Chemotherapy and supportive care

Treatment protocols were adapted from the RATIFY protocol.^12^ Briefly, 7+3 induction consisted of intravenous (IV) cytarabine 200 mg/m^2^ per day by continuous infusion on days 1–7 with IV idarubicin 12 mg/m^2^ per day on days 1–3. Midostaurin 50 mg twice daily (BD) was added to those with *FLT3* mutation from days 8–21. Cytarabine was reduced to 100 mg/m^2^ per day for patients aged ≥ 65 years and/or for those presenting with concurrent severe illness (e.g., sepsis). Those not in CR post‐induction #1 were to receive repeat 7+3 with midostaurin if *FLT3*‐mutated or a HOVON intensified protocol (IV cytarabine 1 g/m^2^ BD on days 1–6 and IV amsacrine 120 mg/m^2^ per day on days 4–6; if age < 60 years) if *FLT3* wild‐type.^21^ The latter regimen was also considered for *FLT3* wild‐type patients already in CR post‐induction #1 if they had adverse ELN risk. Patients achieving CR were planned for three to four cycles of high‐dose cytarabine 3 g/m^2^ BD × 3 days (with dose reductions to 1 g/m^2^ BD in patients aged ≥60 years) plus midostaurin for *FLT3‐*mutated patients. Inpatient consolidations proceeded when the absolute neutrophil count (ANC) was ≥1.0 × 10^9^/L and platelets ≥100 × 10^9^/L. Granulocyte colony‐stimulating factor (G‐CSF) was routinely administered from day 8 until ANC recovery. Anti‐microbial prophylaxis included posaconazole (with a target trough level >0.7 mg/L) and valaciclovir 500 mg BD. Quinolone prophylaxis was not prescribed. Midostaurin dose reductions were only performed in posaconazole‐treated patients in the context of suspected drug‐related adverse events (AEs), without initial adjustments based on patient age. *FLT3*‐mutated patients also received 12 cycles of midostaurin maintenance following completion of consolidation (or until the time of allogeneic stem cell transplantation (allograft)).

### Allograft considerations

Allograft in the first CR (CR1) with complete or incomplete count recovery (CRi) was recommended for all patients with a suitable donor and either adverse‐risk disease or intermediate‐risk disease with an unfavourable measurable residual disease (MRD) response (e.g., failure to attain ELN‐defined MRD landmarks). For the latter, this was typically assessed in alignment with the ELN guidelines, either using real‐time quantitative polymerase chain reaction (sensitivity 10^−5^ to 10^−6^) in those with a leukaemia‐specific marker available (e.g., *NPM1*, *RUNX1::RUNX1T1*, *CBFB::MYH11*) or multi‐parameter flow cytometry (sensitivity 10^−3^ to 10^−4^) in those without, where leukaemia‐associated immunophenotyping was undertaken instead.^22^, ^23^ Favourable and intermediate‐risk patients with MRD negativity per the ELN criteria were not considered for CR1 allograft.^22^, ^23^ All allografts were performed at a separate institution with transfer of care at the time of planned conditioning.

### Response assessments

Bone marrow examinations were performed after all induction and consolidation cycles, and then serially (e.g., every 3 months) following completion of the intensive treatment program for the purpose of MRD monitoring where an appropriate marker was available.^22^, ^23^ Response assessment was according to ELN 2017.^20^ A composite CR (cCR) was defined as CR plus CR with CRi. CRi or morphological leukaemia‐free states were upgraded to CR if ANC and platelet counts reached appropriate thresholds (ANC ≥1.0 × 10^9^/L and platelets ≥100 × 10^9^/L) within 2 weeks of the bone marrow examination. Event‐free survival (EFS) was defined as the duration from initial diagnosis to primary refractory disease, haematologic relapse or death from any cause and censored at last follow‐up. Patients leaving the network or withdrawing consent for further treatment while in CR were censored at the date of departure or withdrawal. OS was defined as the duration from initial diagnosis to death from any cause.

### Safety and toxicity

Duration of neutropenia and thrombocytopenia were defined as days with ANC <1.0 × 10^9^/L and platelets <100 × 10^9^/L during each chemotherapy cycle; for those with neutropenia and/or thrombocytopenia at diagnosis, the onset of cytopenia was set at the first day of induction.^20^ Midostaurin and total parenteral nutrition (TPN) exposure were obtained from electronic and pharmacy dispensing reports. AEs of special interest in midostaurin‐treated patients included gastrointestinal (GI) toxicity, QTc prolongation and unexplained pulmonary infiltrates and graded according to Common Terminology Criteria for Adverse Events version 5.0.

### Statistical analysis

Descriptive statistics of patient characteristics were generated as proportions and medians with ranges for categorical and continuous variables respectively; these were compared between *FLT3‐*mutated versus *FLT3‐*wild‐type subgroups using χ^2^ and Mann–Whitney *U* tests, as appropriate. Median follow‐up was determined by reversing the censor indicator in the Kaplan–Meier analysis of OS.^24^ Survival estimates were assessed by the Kaplan–Meier method and compared using the log‐rank test. A two‐sided *P*‐value <0.05 was considered statistically significant. All statistical analyses and graphical outputs were performed using STATA/MP 14.1 (StataCorp, TX, USA) and GraphPad Prism 10.1.1 (GraphPad Software, MA, USA).

## Results

### Patient characteristics

**Figure 1:**
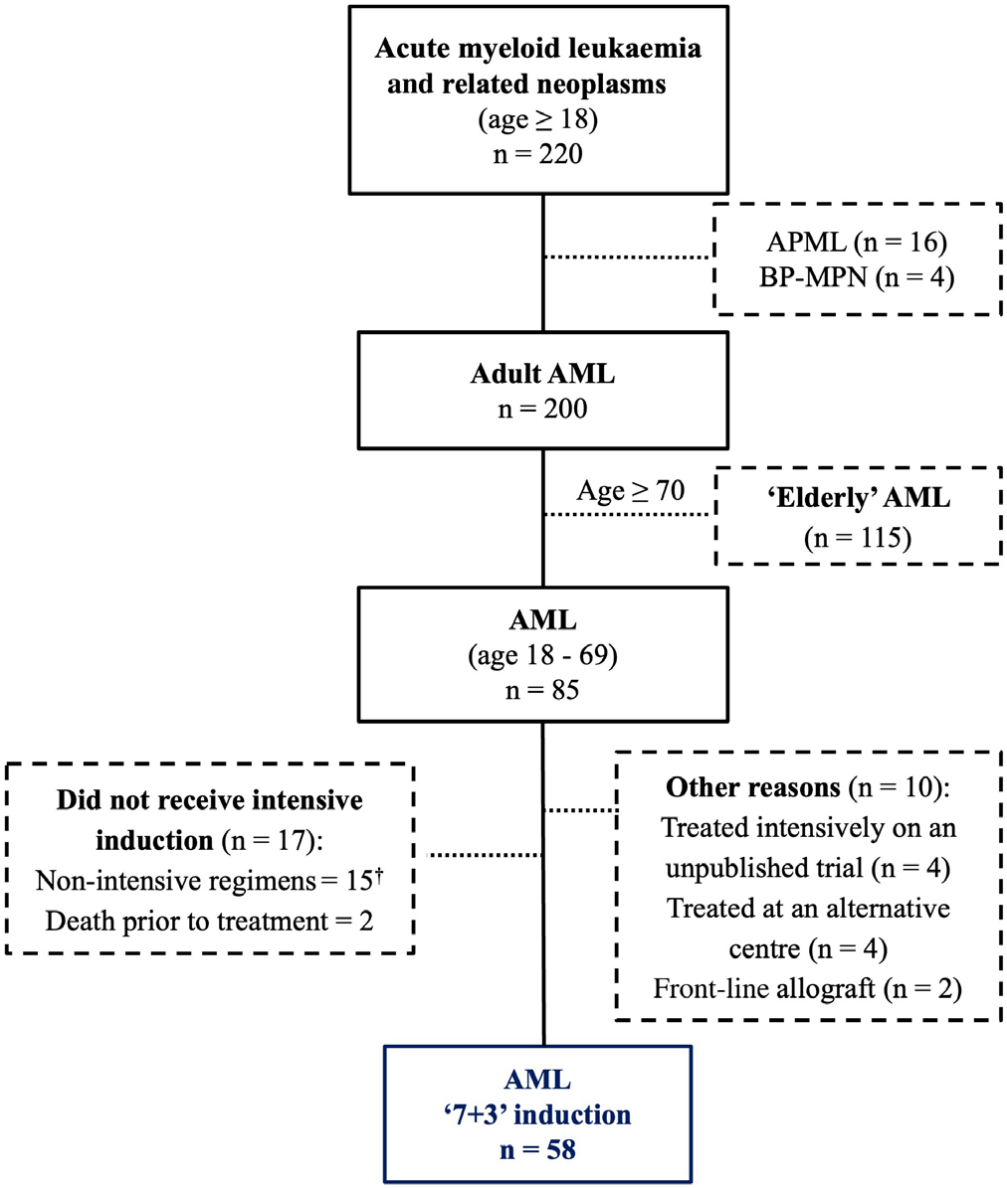
CONSORT (Consolidation Standards of Reporting Trials) diagram of all adult patients presenting with acute myeloid leukaemia during the 5‐year study period (01 August 2018–31 July 2023).^†^ See Table S1 for a clinical summary of therapy and outcomes of these patients. AML, acute myeloid leukaemia; APML, acute promyelocytic leukaemia; BP‐MPN, blast‐phase myeloproliferative neoplasm.

During the 5‐year study period, 220 consecutive adults presented with a new diagnosis of AML and related neoplasms, of whom 58 (26%) received 7+3 induction (Fig. 1). Four additional patients enrolled in clinical trials utilising 7+3 induction were excluded as they received daunorubicin‐based induction and/or investigational agents. The main reason for withholding intensive induction was age ≥ 70 years (*n* = 115). A further 17 patients aged <70 years were not treated intensively: 15 received non‐intensive regimens including venetoclax‐azacitidine (VEN‐AZA, Table S1) and two died of leukaemic complications prior to therapy initiation. Demographics of patients receiving 7+3 induction are presented in Table 1. Median age was 49 years (range: 21–69 years); 41% were female. ELN risk stratification was favourable in 35%, intermediate in 36% and adverse in 29%. *FLT3* mutations were present in 33% of patients (28% ITD and 5% TKD), who presented more frequently with *NPM1* co‐mutation (47% vs 10%, *P* = 0.001) and a significantly higher median white blood cell count (48.6 vs 5.1 × 10^9^/L, *P* = 0.003) compared to *FLT3*‐wild‐type patients. A detailed summary of molecular and cytogenetic characteristics for the derivation of the ELN 2017 risk group is provided in Table S2.

**Table 1:** Baseline patient demographics and disease characteristics of all patients receiving induction therapy and stratified by *FLT3* mutation status

Characteristic	Total (*N* = 58)	*FLT3* ^WT^ (*n* = 39)	*FLT3* ^Mutated^ (*n* = 19)	*P‐value*
Age, years				
Median (range)	49 (21–69)	48 (21–69)	50 (23–69)	0.823
>60, *n* (%)	13 (22)	10 (26)	3 (16)	0.398
Female, *n* (%)	24 (41)	12 (31)	12 (63)	0.019†
AML type, *n* (%)				0.103
*De novo*	53 (91)	34 (87)	19 (100)	
Secondary‡	2 (4)	2 (5)	0 (0)	
Therapy‐related	3 (5)	3 (8)	0 (0)	
PB counts, median (range)				
Hb, g/L	93 (54–150)	91 (54–150)	98 (56–133)	0.607
WBC count, ×10^9^/L	12.8 (0.5–227.7)	5.1 (0.5–227.7)	48.6 (0.9–179)	0.003†
Platelets, ×10^9^/L	61 (6–894)	52 (6–325)	70 (18–894)	0.098
BM blasts, %	71 (13–98)	66 (13–97)	76 (23–98)	0.104
ELN 2017 risk¶, *n* (%)				0.209
Favourable	20 (35)	11 (28)	9 (47)	
Intermediate	21 (36)	14 (36)	7 (37)	
Adverse	17 (29)	14 (36)	3 (16)	
Somatic mutations, *n* (%)				
*NPM1*	13 (22)	4 (10)	9 (47)	0.001†
*FLT3* §	19 (33)	0	19	
*FLT3*‐ITD (AR < 0.50)			12	
*FLT3*‐ITD (AR ≥ 0.50)			4	
*FLT3*‐TKD			3	

† Indicates *P*‐values with statistical significance.

‡ Secondary cases include those with an antecedent histological diagnosis of myelodysplastic syndrome or myelodysplastic‐myeloproliferative neoplasm overlap syndromes.

§ See Table S2 for further details on baseline cytogenetic‐molecular work‐up of patients.

¶ *FLT3* mutations at allelic ratio ≥ 0.01.

AML, acute myeloid leukaemia; BM, bone marrow; ELN, European LeukaemiaNet; *FLT3*, fms‐like tyrosine kinase 3; Hb, haemoglobin; ITD, internal tandem duplication; PB, peripheral blood; TKD, tyrosine kinase domain; WBC, white blood cell; WT, wild‐type.

### Treatment exposure and efficacy outcomes

**Figure 2:**
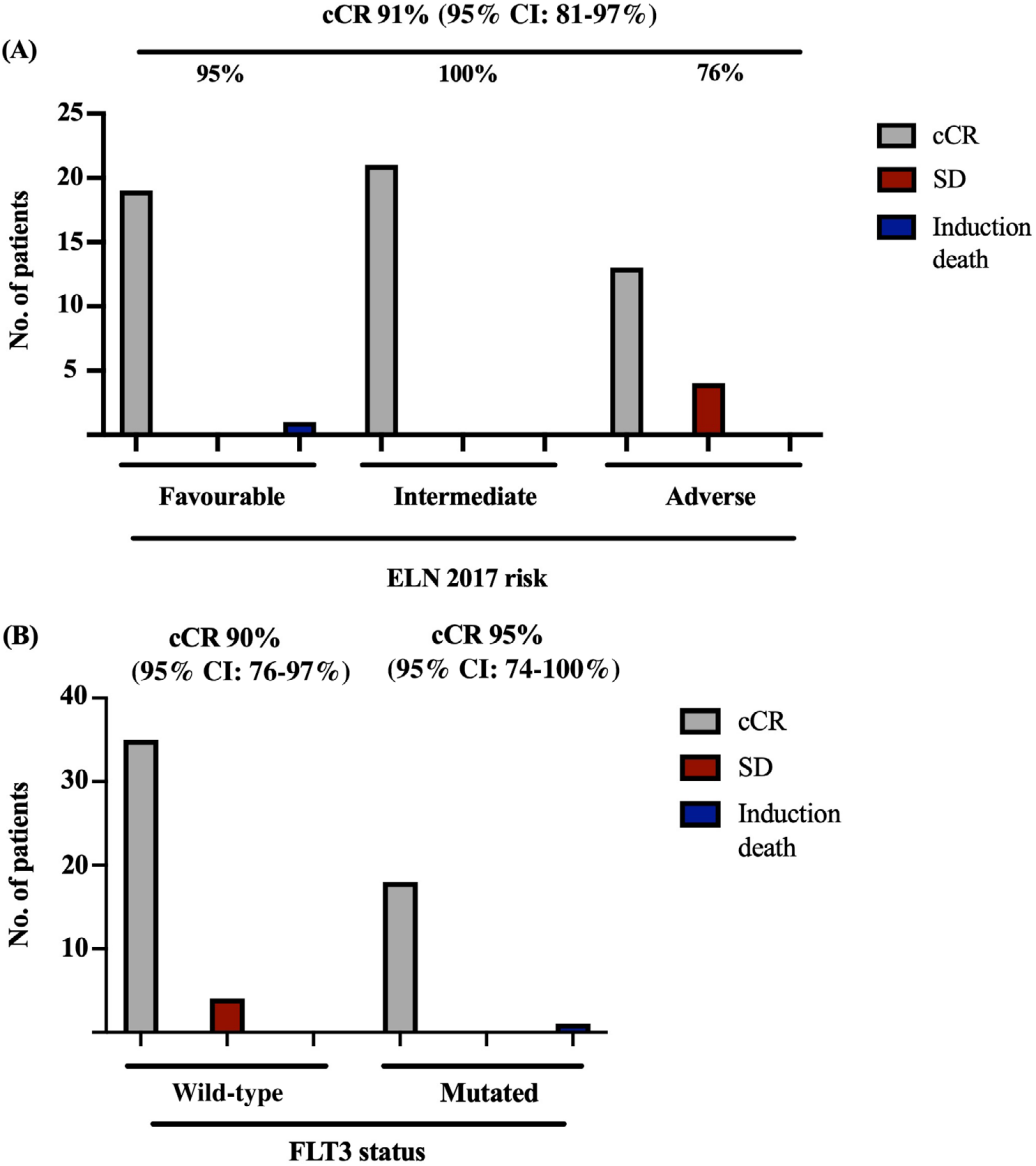
Induction outcomes stratified by (A) ELN 2017 risk classification and (B) *FLT3* mutation status. The percentage of cCRs are provided at the top of each panel. cCR, composite complete remission (complete remission + complete remission with incomplete count recovery); ELN, European LeukaemiaNet; *FLT3*, fms‐like tyrosine kinase 3; SD, stable disease.

Following induction, the cCR rate was 91% (95% confidence interval (95% CI): 81–97%, Table 2), consisting of 51 CRs (88%, 95% CI: 77–95%) and two CRi, which were all achieved following induction #1. Patients with adverse‐risk AML were less likely to enter cCR than those with non‐adverse risk (76% vs 98%, *P* = 0.009), whereas *FLT3* mutation status did not impact cCR attainment (95% *FLT3‐*mutated vs 90% *FLT3* wild‐type, *P* = 0.525; Fig. 2). With one CRi response in the *FLT3*‐mutated subgroup, the CR rate was 89% (95% CI: 67–99%). There was one induction death due to septic shock prior to response evaluation. Four patients (7%) did not attain cCR post induction #1; all of whom had adverse‐risk disease and none received a second round of intensive induction. Four patients (7%) in CR received a second induction (Table 2). Detailed descriptions of patient disposition following induction and consolidation phases are provided in Figure S1.

**Table 2:** Chemotherapy courses received and induction responses

Treatment details	Total
Induction phase	(*N* = 58)
Induction #1, *n* (%)	
7+3 (Ara‐C 200 mg/m^2^)	46 (79)
7+3 (Ara‐C 100 mg/m^2^)	12 (21)
Additional targeted therapy	19 (34)
Midostaurin†	16 (28)
Others‡	3 (5)
Induction #2§, *n* (%)	4 (7)
7+3 (Ara‐C 200 mg/m^2^)	2 (3)
ID‐Ara‐C + Amsacrine	2 (3)
Induction response, *n* (%)	
CR	51 (88)
CRi¶	2 (3)
MLFS	0 (0)
PR	0 (0)
SD	4 (7)
Induction death	1 (1.7)

Post‐induction remission phase	(*n* = 53)
HD‐Ara‐C consolidation, *n* (%)	45 (85)
One cycle	7 (13)
Two cycles	6 (11)
Three cycles	16 (30)
Four cycles	16 (30)
Allogeneic SCT in CR1	15 (28)
Baseline adverse ELN risk	9 (17)
Intermediate ELN risk + unfavourable MRD	5 (9)
Intermediate ELN risk + therapy‐related AML	1 (2)

† Three patients with *FLT3* mutations did not receive *FLT3* inhibitors due to concurrent severe illness during induction.

‡ Two patients received gemtuzumab ozogamicin for CD33+ core‐binding factor AML; one patient received dasatinib for BCR:ABL1‐positive AML.

§ All four patients prescribed induction #2 were in CR following induction #1; reasons for induction #2 include baseline adverse ELN risk and pre‐specified double induction plan (*n* = 2), intermediate ELN risk with unfavourable MRD (*n* = 1) and truncated induction #1 due to toxicity (*n* = 1).

¶ CRi in two patients due to incomplete platelet recovery prior to next course of therapy.

AML, acute myeloid leukaemia; Ara‐C, cytarabine; CR, complete remission; CRi, complete remission with incomplete count recovery; CR1, first complete remission; ELN, European LeukaemiaNet; *FLT3*, fms‐like tyrosine kinase 3; HD, high‐dose; ID, intermediate dose; MLFS, morphologic leukaemia‐free state; MRD, measurable residual disease; PR, partial remission; SCT, stem cell transplant; SD, stable disease.

Of the 53 patients in CR1 after induction, 45 (85%) received at least one high‐dose cytarabine consolidation cycle (median = 3) and 15 (28%) proceeded to CR1 allograft (Table 2) after a median of two consolidations (Fig. S1). Amongst the 17 patients with adverse baseline ELN risk, nine were allografted in CR1, yielding an allograft realisation rate of 53%. The rest were not allografted due to primary refractory disease (*n* = 4), haematologic relapse prior to allograft (*n* = 2), preclusive treatment‐emergent renal impairment (*n* = 1) and early and sustained attainment of CR with MRD negativity (*n* = 1). With a median followup of 24.6 months at data censoring, 18 out of 58 patients (31%) had died from the following causes: refractory/relapsed disease (22%), induction death (1.7%) and treatment‐related complications (7%; three post allograft and one during salvage therapy). Nine further patients (16%) had relapsed but were alive at the time of analysis.

**Figure 3:**
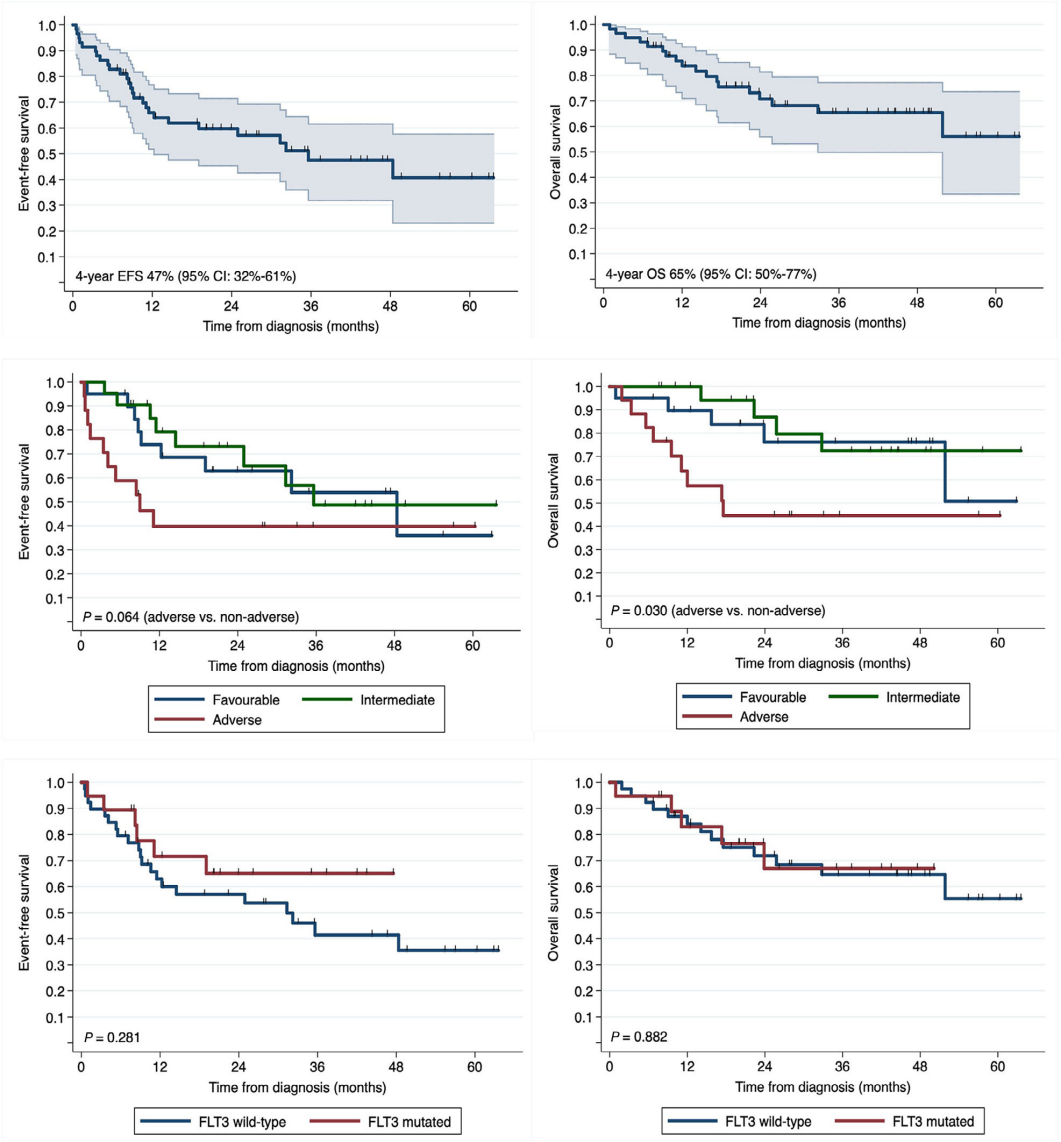
EFS (left panel) and OS (right panel) for the whole cohort (top row) and stratified by ELN 2017 risk classification (middle row) and by *FLT3* mutation status (bottom row). EFS, event‐free survival; ELN, European LeukaemiaNet; *FLT3*, fms‐like tyrosine kinase 3; OS, overall survival.

Survival data are summarised in Figure 3. The median EFS was 35.6 months (95% CI: 12.3 – not reached) with a median OS not reached (4‐year OS 65%; 95% CI: 50–77%). Adverse ELN risk AML portended worse EFS (*P* = 0.064; median 9 months) and OS (*P* = 0.030; median 17.6 months) compared to their non‐adverse counterparts. In the *FLT3‐*mutated subgroup, the median EFS and OS were not reached, with a 4‐year EFS of 65% (95% CI: 37–83%) and OS of 68% (95% CI: 37–86%); which were comparable to those of the *FLT3* wild‐type subgroup (Fig. 3, bottom panel). Six patients with *FLT3* mutation were allografted in CR1, while four patients (21%) were allografted following either a haematologic (*n* = 3) or molecular (*n* = 1) relapse.

### Safety and resource utilisation

The median time to neutrophil and platelet recovery following induction for patients attaining CR was 22 days (range: 15–33 days) and 27 days (range: 20–86 days), respectively (Fig. S2). The median duration of neutropenia during consolidation cycle one was 9 days (range: 4–23 days) and 15 days (range: 7–23 days) for consolidation cycle four. The median duration of thrombocytopenia was 16 days (range: 8–47 days) for consolidation cycle one and 30 days (range: 16–57) for consolidation cycle four. The median inpatient length of stay was 30 days (range: 22–75 days) for induction, 20 days (range: 12–29 days) for consolidation cycle one and 22 days (range: 14–29 days) for consolidation cycle four. Intensive care unit (ICU) support was required for 21% of patients (95% CI: 11–33%) during induction and none during consolidation four. Lower rates of ICU admission were observed across consolidation cycles one to three (range: 3–11%), with none in consolidation four; all episodes were of 5 days' duration or less. There were no deaths over 169 consolidation cycles. TPN was required exclusively during induction (eight patients, 14%; median duration = 8 days, range: 3–24 days); specific data on mucositis rates were not recorded.

### Midostaurin treatment

For the 19 patients with *FLT3‐*mutated AML (Table 3), three were not prescribed midostaurin during induction due to pre‐existing grade 3 mucositis in one and delayed detection of *FLT3* mutation in two. Of the 16 patients who received midostaurin therapy, only about one‐quarter received the full intended dose (50 mg BD × 14 days; 1400 mg). The most common reason for withholding midostaurin was GI toxicity (seven patients, 44%; TPN‐requiring grade 3 toxicity in three (19%) on clinical (diarrhoea) and/or radiological grounds (e.g., computed tomography evidence of colitis)), followed by QTc prolongation (three patients, 19%). All 16 patients received netupitant (with palonosetron) as anti‐emetic during induction and posaconazole prophylaxis, with a median posaconazole level of 0.67 mg/L, while other QTc‐prolonging agents were used sparingly during induction (median = two medications). There was higher overall delivery of midostaurin during consolidation, where the majority received the full dose (range: 65%–83%) or between half and the full dose (range: 17%–29%). Additional AEs of interest during consolidation included non‐arrhythmic cardiac toxicity (one patient with ST depression, another with myocarditis) with a case of grade 3 QTc prolongation but no grade 3 GI toxicity. Across all inductions and consolidations, there were no unexplained pulmonary infiltrates considered related to midostaurin. Higher rates of concomitant QTc‐prolonging medication use were noted during consolidation compared to induction (median = three or four vs two medications during induction), particularly of anti‐emetics and, to a lesser extent, anti‐microbials and analgesics (Table 3 and Table S3).

**Table 3:** Real‐world clinical practice and AEs associated with midostaurin use in the setting of 7+3 idarubicin induction

Characteristics	Induction (*n* = 19)	Cons #1 (*n* = 17)	Cons #2 (*n* = 15)	Cons #3 (*n* = 12)	Cons #4 (*n* = 6)
Midostaurin dose exposure, *n* (%)					
Omitted	3 (16)	0 (0)	0 (0)	1 (8)	0
<700 mg	4 (21)	1 (6)	0 (0)	0	0
700–1350 mg	7 (37)	5 (29)	3 (20)	2 (17)	1 (17)
Full dose (1400 mg)	5 (26)	11 (65)	12 (80)	9 (75)	5 (83)

Concomitant medications‡,§	*(n = 16)*	*(n = 17)*	*(n = 15)*	*(n = 11)*	*(n = 6)*
CYP3A4 inhibitors					
Posaconazole prophylaxis¶, *n* (%)	16 (100)	16 (94)††	15 (100)	10 (91)††	6 (100)
Median level, mg/L (range)	0.67 (0.21–2.10)				
Median timing of level, days post‐induction (range)	12 (8–23)				
Netupitant (as Akynzeo), *n* (%)	16 (100)	17 (100)	15 (100)	11 (100)	5 (83)
QTc‐prolonging agents, *n* (%)					
Anti‐microbials, *n* (%)					
Azithromycin	1 (6)	1 (6)	0 (0)	1 (9)	0 (0)
Ciprofloxacin	1 (6)	1 (6)	3 (20)	2 (18)	1 (17)
Remdesivir	0 (0)	0 (0)	1 (7)	0 (0)	0 (0)
Anti‐emetics, *n* (%)					
Chlorpromazine	0 (0)	0 (0)	1 (7)	0 (0)	0 (0)
Metoclopramide	0 (0)	10 (59)	9 (60)	6 (55)	2 (33)
Ondansetron	1 (6)	5 (29)	5 (33)	2 (18)	2 (33)
Others, *n* (%)					
Fluoxetine	1 (6)	1 (6)	1 (7)	1 (9)	0 (0)
Tramadol	1 (6)	1 (6)	4 (27)	1 (9)	0 (0)
Ivabradine	0 (0)	1 (6)	0 (0)	0 (0)	0 (0)
Median no. of meds per patient, (range)	2 (2–4)	3 (2–5)	4 (2–6)	3 (2–5)	3 (2–4)

AEs of interest	*(n = 16)*	*(n = 17)*	*(n = 15)*	*(n = 11)*	*(n = 6)*
Diarrhoea (any grade), *n* (%)	14 (88)	6 (35)	7 (47)	6 (55)	2 (33)
TPN requirement (grade 3)	3 (19)	0 (0)	0 (0)	0 (0)	0 (0)
QTcF prolongation†, *n* (%)					
No significant change	1/9 (11)	5/9 (56)	7/9 (78)	5/7 (71)	2/3 (67)
New onset 450–480 msec	5/9‡‡ (56)	3/9 (33)	2/9 (22)	0/7 (0)	0/3 (0)
New onset 480–500 msec	0/9 (0)	0/9 (0)	0/9 (0)	0/7 (0)	0/3 (0)
New onset >500 msec	0/9 (0)	0/9 (0)	0/9 (0)	1/7‡‡ (14)	0/3 (0)
>30 msec from baseline	5/9 (56)	2/9 (22)	0/9 (0)	2/7 (29)	1/3 (33)
>60 msec from baseline	2/9 (22)	0/9 (0)	0/9 (0)	0/7 (0)	0/3 (0)

† Patients with at least one retrievable electrocardiographic recording prior to plus during midostaurin use only were included; the number of evaluable patients is therefore different each cycle. QTcFs were calculated by Fridericia formulae. One patient may have more than one cardiac rhythm change, e.g., >30 msec from baseline and new onset >450–480 msec.

‡ The denominator was set as the number of patients receiving at least one dose of midostaurin for each cycle.

§ See Table S3 for a full list of medications with CYP3A4 inhibitory and/or QT interval‐interfering properties utilised by patients while concurrently taking midostaurin.

¶ Posaconazole also has QT‐prolonging properties. Posaconazole levels recorded were the highest trough level measured (if more than one measurement) during induction and after at least 5 days of intended prophylactic dose exposure; these were available in all but one patient.

†† Alternative anti‐fungal prophylaxis was provided in the form of liposomal amphotericin B.

‡‡ One of these patients had a prolonged baseline QTcF of 481 msec, with QTcF of 479 msec while taking midostaurin during induction. The same patient had a baseline QTcF of 459 msec during consolidation 3, which went up to 518 msec without onset of Torsades de Pointes.

AE, adverse event; Cons, consolidation; CYP3A4, cytochrome P450 enzyme; QTcF, corrected QT interval (Fridericia formula); TPN, total parenteral nutrition.

## Discussion

Real‐world AML series often yield poorer outcomes than the clinical trial comparator on which they are based, which may reflect divergence from protocolised management and/or inclusion of patients who would have been excluded from trial entry (e.g., hyperleukocytosis, poor performance status).^3^ Here, we describe the application of ALLG consensus recommendations for idarubicin‐based 7+3 induction with the addition of midostaurin for *FLT3*‐mutant AML. With contemporary supportive care, treatment‐related mortality was low. Remission rates, EFS and OS for the cohort benchmarked well against published trial results and recently published outcomes from the ALLG National Blood Cancer Registry (NBCR).^9^, ^12^, ^25^ Patients with *FLT3* mutations had comparable outcomes to those without, thereby supporting the shift in baseline allocation of *FLT3‐*ITD mutation with a high AR from ‘adverse’ to ‘intermediate’ risk in the current ELN schema.^1^ By contrast, outcomes with adverse ELN risk AML remained suboptimal with a 7+3‐based regimen.

The delivery of intensive AML chemotherapy is resource‐heavy, as reflected in a 30‐day median length of induction inpatient stay. However, improved safety was highlighted by a low induction death rate. This may be explained by advances in supportive care, including routine use of mould‐active prophylaxis^6^ as well as routine use of early G‐CSF; the latter resulting in an apparent shorter duration of neutropenia in our cohort relative to other series, such as the RADIUS‐X expanded access midostaurin program (median 22 vs 26 days).^26^ Consolidations were well tolerated, with no deaths or TPN requirement and low ICU admission rates. Importantly, despite treatment‐limiting thrombocytopenia in some patients, 60% of the cohort eligible for consolidation (i.e., in CR post‐induction) received three or more cytarabine cycles, while another quarter received fewer than three cycles due to an allograft in CR1. These data suggest that adequate front‐line anti‐leukaemic treatment had been received by most patients.^27^

Several observations emerged in patients with *FLT3*‐mutant AML in the context of midostaurin therapy.^25^ The 89% CR rate for this subgroup compares favourably to the 59% CR rate in the midostaurin arm of the RATIFY trial and 60% CR rate for RATIFY‐eligible patients entered into the ALLG NBCR, with comparable 4‐year EFS and OS (65% and 68% respectively in this study).^12^, ^25^ A likely contributor to the high CR rate was a greater proportion of patients with *FLT3* mutation in our cohort with favourable risk AML.^28^ In the context of idarubicin use as our backbone anthracycline, the higher prevalence of GI toxicity in our midostaurin‐exposed cohort compared to the RADIUS‐X idarubicin cohort, where azole prophylaxis was not mandatory, may indicate an effect of posaconazole boosting midostaurin levels (maximum median level as high as 2.10 mg/L in our cohort), and hence poorer tolerability.^26^ Similar observations were noted against the idarubicin cohort reported by Sierra and colleagues (*n* = 166, only 75% were prescribed concurrent anti‐fungals; drug levels not reported), where cumulative grade ≥ 3 GI toxicity was notably only around 3% (vs 19% in our study); although here, 20% of the cohort did receive the less intensive 5+2 induction chemotherapy also.^29^ Another relevant observation in our study was the relatively common occurrence of QTc prolongation during induction, as also observed in Sierra *et al*.^29^ Multiple concomitant medications with QTc‐prolonging properties were found to be used liberally throughout the treatment course. These findings highlight the need for heightened pharmaco‐vigilance (e.g., close therapeutic drug monitoring) throughout treatment to prevent AEs. Alternatively, as has recently been shown in the German‐Austrian AML Study Group 16–10, dose reductions amidst concurrent strong CYP3A4 inhibitors (Table S3) could also be considered upfront (e.g., 25 mg every other day) without compromising treatment efficacy.^30^

Patients with adverse‐risk AML remain a subgroup with an unmet need. Survival outcomes were poor, underpinned by suboptimal induction responses and failure to maintain remission. A key priority for such patients is maintaining both performance status and disease control to permit potentially curative allograft, which was realised in only one‐half of our adverse‐risk patients.^1^ Thus, the benefit‐to‐toxicity ratio of potentially futile intensive chemotherapy should be carefully considered. This was reflected in our divergence from intensification by second induction, or a pivot to low‐intensity VEN‐AZA inductions for adverse‐risk patients after PBS approval (in December 2021) of this alternative. Although not the focus of this manuscript, it is notable that four out of six adverse‐risk patients aged <70 years who received first‐line VEN‐AZA instead of 7+3 (Table S1) and a further two of four salvaged for refractory disease attained CR and were successfully allografted.

Several caveats to our study merit comment. First, while conducted prospectively with a uniform approach to treatment, our study was restricted to a single network with a relatively small sample size and short follow‐up. Second, the ALLG consensus approach to AML management remains dynamic to respond to key therapeutic developments. To this end, the preference for single versus (routine) double induction remains contentious^31^; where the latter is standard practice in some regions (e.g., the United Kingdom^9^) but as apparent in our cohort, may not be necessary to attain favourable outcomes in many patients. Moreover, different chemotherapy backbones may have selective benefit in molecular subgroups such that a one size‐fits‐all ‘7+3‐like induction’ is likely to be an oversimplified model.^32^

## Conclusion

In summary, the application of ALLG consensus recommendations featuring the inclusion of PBS‐reimbursed midostaurin was associated with acceptable safety and efficacy in our cohort of 58 patients with newly diagnosed AML, benchmarking favourably against published trial and registry data. *FLT3*‐mutant AML had excellent CR rates and long‐term survival comparable to the RATIFY trial comparator cohort attained with substitution of daunorubicin with idarubicin. In contrast, patients with adverse ELN risk AML fared poorly, requiring new approaches including consideration for VEN‐AZA‐based induction.

## Supporting information


**Data S1** Supporting Information.
