# Supplementary material for: Ratifying the efficacy and safety of intensive induction chemotherapy for acute myeloid leukaemia by the Australasian Leukaemia & Lymphoma Group consensus approach
Source: Intern Med J. 2025 Mar 7;55(5):749–59. doi: 10.1111/imj.70010 (PMC12077584; doi:10.1111/imj.70010)
Supplement: Supplementary file 1 — Data S1 Supporting Information. [file IMJ-55-749-s001.docx]

**Supplementary materials to:**

A Tedjaseputra et al.

**‘Ratifying the efficacy and safety of intensive induction chemotherapy for acute myeloid leukaemia by the Australasian Leukaemia and Lymphoma Group consensus approach’**

**Table S1.** Summary or patients with AML aged 18-69 who received non-intensive induction therapy and related outcomes.

**Table S2.** Baseline cytogenetics and gene mutation results of all patients for assignment into the ELN 2017 risk stratification.

**Table S3.** Full list of concomitant medications with significant drug-drug interaction with midostaurin of potential clinical significance.

**Figure S1.** Patient flow through intensive induction and consolidation chemotherapy by cycle of treatment.

**Figure S2.** Duration of cytopenia and length of stay per induction and consolidation cycles: duration of (A) neutropenia (ANC < 1.0 x 10^9^/L) and (B) thrombocytopenia (PLT < 100 x 10^9^/L) and length of (C) inpatient hospital stay and (D) ICU stay. Number of patients per consolidation cycle is given below each corresponding dot plot.

**Table S1**. Summary or patients with AML aged 18-69 who received non-intensive induction therapy and related outcomes.

| Patient | Age  Sex | PS | Major co-morbidities | Nature of AML | Cytogenetic profile | Gene mutations | ELN 2017  risk strata | Rationale for non-intensive induction | Treatment | CR1  achieved | Date of  diagnosis |
| --- | --- | --- | --- | --- | --- | --- | --- | --- | --- | --- | --- |
| PRE-Venetoclax PBS listing^†^ | | | | | | | | | | | |
| 1 | 67M | 1 | MAFLD  (non-cirrhotic) | De novo | Complex & monosomal | No mutations | Adverse | [1] Predicted poor response to conventional intensive chemotherapy | AZA | No | Oct ‘19 |
| 2 | 66M | 0 | Nil | Likely secondary**^‡^** (likely prior CMML) | Normal | *ASXL1, SRSF2, TET2* | Adverse | [1] Patient preference and adverse mutational profile | AZA | Yes | Jul ‘20 |
| 3 | 46M | 4 | Schizophrenia | De novo | Trisomy 11 | *FLT3-ITD* (AR 0.26), *IDH2* | Intermediate | [1] Unfit due to co-morbidities at presentation (catatonic schizophrenia) | LDAC+TG | Yes | Aug ‘20 |
| 4 | 66M | 2 | PVD, COPD | De novo | Complex & monosomal | No mutations | Adverse | [1] Predicted poor response to conventional intensive chemotherapy  [2] Unfit due to co-morbidities at presentation (septic shock) | AZA | No | Nov ‘20 |
| 5 | 52M | 2 | HL in remission (prior chemo- radiotherapy) | Therapy-related | Complex & monosomal | *IDH1, TP53* | Adverse | [1] Predicted poor response to conventional intensive chemotherapy  [2] Predicted poor tolerability of intensive chemotherapy | AZA | No | Nov ‘20 |
| 6 | 69M | 2 | IHD, recent stroke | De novo | t(6;9) DEK::NUP214 | *FLT3-ITD* (AR 0.37) | Adverse | [1] Unfit due to co-morbidities at presentation (cardiovascular disease) | LDAC+TG | No | Dec ‘20 |
| 7 | 64M | 1 | HL in remission (prior chemotherapy) COPD | Therapy-related | Monosomy 7 | *FLT3*-ITD (AR 0.86) | Adverse | 1] Predicted poor response to conventional intensive chemotherapy  [2] Unfit due to co-morbidities at presentation (advanced lung disease) | AZA + BCL2 inhibitor | N/A^§^ | Sep ‘21 |
| POST-Venetoclax PBS listing^†^ | | | | | | | | | | | |
| 8 | 64M | 0 | OSA | De novo | Abnormal 7q, marker chromosome | *DNMT3A, RUNX1, SRSF2, TET2* | Adverse | [1] Patient preference  [2] Unfit due to co-morbidities at presentation (chest infection and haemoptysis) | Ven-AZA ⇒ Allo-SCT in CR1 | Yes | Apr’ 22 |
| 9 | 60M | 3 | NHL in remission (prior chemotherapy), cirrhosis | Therapy-related | Complex & monosomal | *TP53*, *ZRSR2* | Adverse | [1] Predicted poor response to conventional intensive chemotherapy  [2] Unfit due to co-morbidities at presentation (cirrhosis and neutropenic sepsis) | Ven-AZA ⇒ Allo-SCT in CR1 | Yes | Sep ‘22 |
| 10 | 61M | 4 | Autoimmune encephalitis | De novo | Normal | *ASXL1, EZH2, RUNX1, TET2, ZRSR2* | Adverse | [1] Predicted poor response to conventional intensive chemotherapy  [2] Unfit due to co-morbidities at presentation (encephalitis) | AZA | No | Nov ‘22 |
| 11 | 65M | 0 | Nil | De novo | Complex & monosomal | *IDH1, PHF6, TP53* | Adverse | [1] Patient preference  [2] Predicted poor response to conventional intensive chemotherapy | Ven-AZA ⇒ Allo-SCT in CR1 | Yes | Jan ‘23 |
| 12 | 59M | 1 | Nil | De novo | Complex & monosomal | *TP53* | Adverse | [1] Predicted poor response to conventional intensive chemotherapy | Ven-AZA | No | Jan ‘23 |
| 13 | 59F | 0 | Nil | Secondary**^‡^** (MDS) | Normal | *DNMT3A, NRAS, RUNX1, SF3B1, TET2* | Adverse | [1] Patient preference | Ven-AZA | No | Feb ‘23 |
| 14 | 65F | 0 | Nil | De novo | Complex & monosomal | *CBL, FLT3, NRAS, TP53* | Adverse | [1] Patient preference  [2] Predicted poor response to conventional intensive chemotherapy | Ven-AZA ⇒ Allo-SCT in CR1 | Yes | Mar ‘23 |
| 15 | 34M | 3 | Bowel cancer (prior chemotherapy) | Therapy-related | Normal | *DNMT3A, NPM1, NRAS* | Favourable | [1] Unfit due to severe co-morbidities at presentation (bowel obstruction and sepsis) | Ven-AZA | Yes | May ‘23 |

^†^ Venetoclax and Azacitidine combination received PBS approval for use in patients with AML unfit for intensive chemotherapy in Australia from December 2021 onwards.

**^‡^** Secondary cases include those diagnosed with an antecedent histological diagnosis of myelodysplastic syndrome or myelodysplastic-myeloproliferative neoplasm overlap syndromes.

^§^ Treated on a yet to be published clinical study; outcome not disclosed.

Allo-SCT, allogeneic stem cell transplantation; AML, acute myeloid leukaemia; AZA, azacitidine; COPD, chronic obstructive pulmonary disease; CR1, 1^st^ complete remission; ELN; European Leukaemia Net; HL, Hodgkin lymphoma; IHD, ischaemic heart disease; LDAC/TG, low dose cytarabine and thioguanine; MAFLD, metabolic associated fatty liver disease; NHL, non-Hodgkin lymphoma; OSA, obstructive sleep apnoea; PS, Performance Status by Eastern Cooperative Oncology Group; PVD, peripheral vascular disease; Ven, venetoclax.

**Table S2.** Baseline cytogenetics and gene mutation results of all patients for assignment into the ELN 2017 risk stratification.

| **Molecular and cytogenetic abnormalities for ELN 2017 risk classification** | **Total**  (n = 58) |
| --- | --- |
| **Complete** | 49 |
| **AML with defining CG abnormalities** | 20 |
| t(8;21)(q22;q22.1) | 1 |
| inv(16)(p13.1q22) | 7 |
| t(9;11)(p21.3;q23.3) | 2 |
| t(6;9)(p23;q34.1) | 1 |
| t(v;11q23.3) | 2 |
| t(9;22)(q34.1(q11.2) | 1 |
| Complex and/or monosomal karyotype | 6 |
| **AML with biallelic *CEBPA* mutations** | 2 |
| **AML with *NPM1* mutation** | 11 |
| *FLT3* unmutated | 3 |
| *FLT3*-ITD with low AR | 6 |
| *FLT3*-ITD with high AR | 2 |
| **AML with *NPM1* wild-type and *FLT3­*-ITD with high AR** | 2 |
| **AML with mutated *RUNX1* and/or *ASXL1*** | 5 |
| **AML with CG abnormalities not classified as favourable or adverse** | 9 |
| **Incomplete (assigned as intermediate ELN 2017 risk)** | 9 |
| Intermediate-risk CG without *CEBPA* analysed | 4 |
| Failed mitoses without *CEBPA* analysed | 1 |
| Intermediate-risk CG without *CEBPA*, *ASXL1*, *RUNX1*, *TP53* analysed | 4 |

AR, allelic ratio; CG, cytogenetics.

**Table S3.** Full list of concomitant medications with significant drug-drug interaction with midostaurin of potential clinical significance.

| **Medication** | **CYP3A4 inhibition**  (⇣ Midostaurin metabolism & PK boosting) | **QTc prolongation risk**  (Concurrent ⇡ in QTc prolongation effect) |
| --- | --- | --- |
| **Azithromycin** | No | Yes |
| **Chlorpromazine** | No | Yes |
| **Ciprofloxacin** | No | Yes |
| **Fluoxetine** | No | Yes^‡^ |
| **Ivabradine** | No | Yes |
| **Metoclopramide** | No | Yes^‡^ |
| **Netupitant**^†^ | Yes (moderate) | No |
| **Palonosetron**^†^ **Ondansetron** | No  No | Yes^‡^  Yes |
| **Posaconazole** | Yes (strong) | Yes^‡^ |
| **Remdesivir** | No | Yes^‡^ |
| **Tramadol** | No | Yes^‡^ |

^†^ These medications were administered to patients during their intensive treatment program; albeit not concurrent with or within 3 days pre- or post-midostaurin prescription.

^‡^ Possible or conditional risk of Torsades de Pointes when used with other QT-prolonging drugs, including midostaurin.

**Figure S1.** Patient flow through intensive induction and consolidation chemotherapy by cycle of treatment.

**HD-AraC**

**consolidation #2**

n = 37

**HD-AraC**

**consolidation #2**

n = 1

**‘7+3’ induction #1**

n = 58

**HD-AraC**

**consolidation #1**

n = 44

**No consolidation (n = 13):**

| 1^0^ refractory AML | 4 |
| --- | --- |
| Excess toxicity ± favourable MRD^†^ | 1 |
| Allo-SCT in CR1 | 5 |
| Induction death | 1 |
| Haematologic relapse | 2 |

**Induction #2**

n = 4

**HD-AraC**

**consolidation #1**

n = 1

**No induction #2**

n = 54

n = 3

n = 10

**1 consolidation only (n = 7):**

| Excess toxicity ± favourable MRD^†^ | 3 |
| --- | --- |
| Allo-SCT in CR1 | 3 |
| Haematologic relapse | 1 |

n = 7

n = 0

**2 consolidations only (n = 6):**

| Excess toxicity ± favourable MRD^†^ | 1 |
| --- | --- |
| Allo-SCT in CR1 | 5 |

n = 6

n = 0

**HD-AraC**

**consolidation #3**

n = 31

**HD-AraC**

**consolidation #4**

n = 16^§^

**HD-AraC**

**consolidation #3**

n = 1

**3 consolidations only**

**(n = 16):**

| Received 2 ‘7+3’ inductions | 1 |
| --- | --- |
| Excess toxicity ± favourable MRD^†^ | 11 |
| Allo-SCT in CR1 | 1 |
| Haematologic relapse | 1 |
| Other^‡^ | 2 |

n = 1

n = 15

^†^ Patients who exited intensive treatment program early were subsequently commenced on various maintenance therapies (e.g. FLT3-inhibitors, clinical study) or underwent routine monitoring with serial FBE, BM and MRD assessments.

^‡^ One patient did not receive the fourth consolidation cycle due to patient preference, and the other due to an unknown reason.

^§^ One further patient received deferred allo-SCT in CR1 due to pregnancy; the patient was successfully transplanted post-delivery while in CR1 with low-level measurable residual disease.

Allo-SCT, allogeneic stem cell transplant; AML, acute myeloid leukaemia; BM, bone marrow; CR1, 1^st^ complete remission; FBE, full blood examination; MRD, measurable residual disease.

**Figure S2.** Duration of cytopenia and length of stay per induction and consolidation cycles: duration of (A) neutropenia (ANC < 1.0 x 10^9^/L) and (B) thrombocytopenia (PLT < 100 x 10^9^/L) and length of (C) inpatient hospital stay and (D) ICU stay. Number of patients per consolidation cycle is given below each corresponding dot plot.


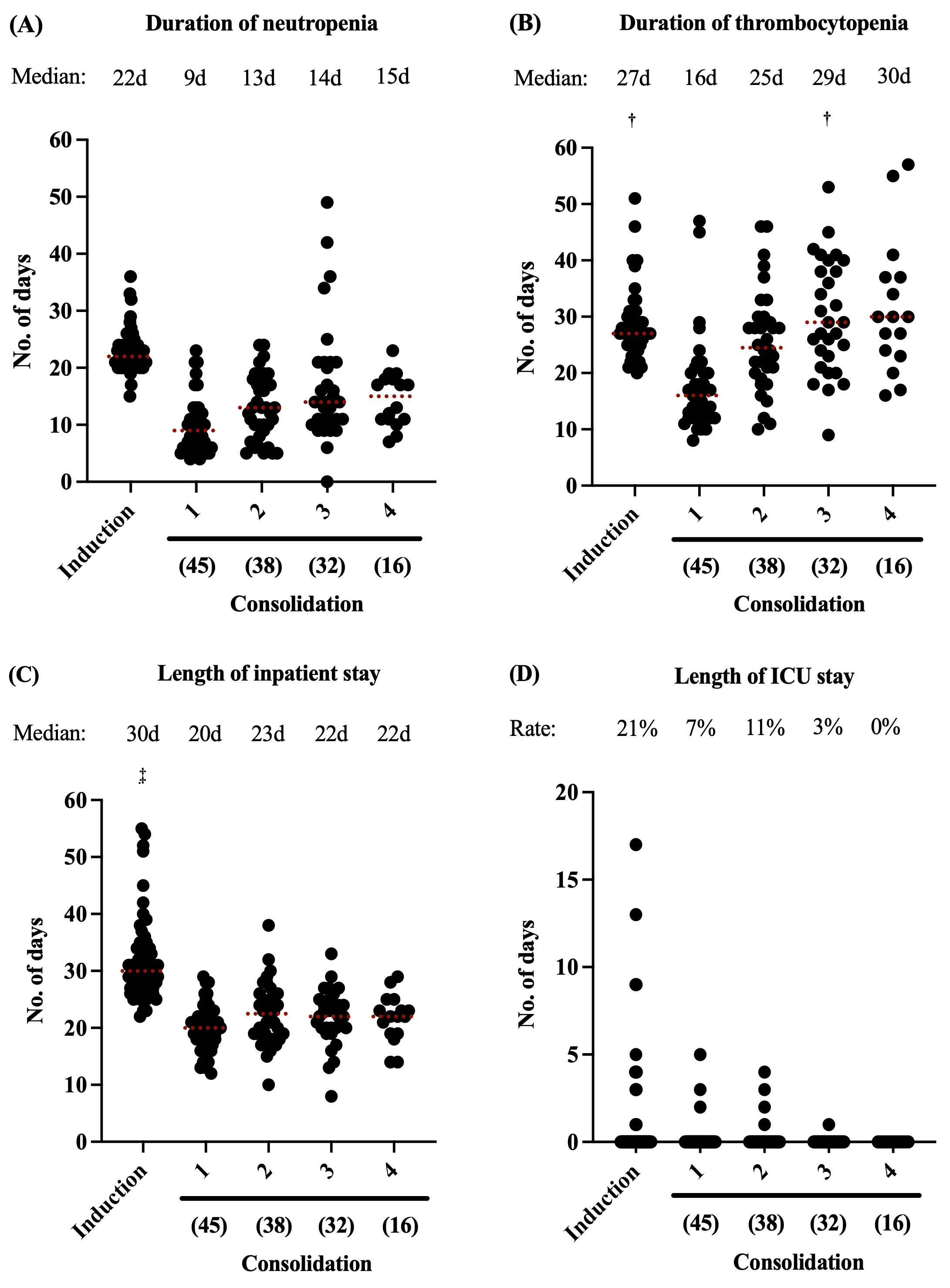


^†^ Denotes one and two data points outside range not displayed for induction (86 days) and consolidation 3 (82 & 107 days), respectively. ^‡^ Denotes one data point outside range not displayed for induction (75 days)
